# Nutritional counselling and risk factors for obesity: an observational study in toddlers

**DOI:** 10.1186/s13052-024-01668-z

**Published:** 2024-06-13

**Authors:** Raffaele Limauro, Luigi Cioffi, Vincenzo Bianco, Vincenzo Caruso, Antonella Casani, Donatella Del Gaizo, Aldo Esposito, Evelina Farris, Patrizia Gallo, Maria Giuliano, Agnese Iannone, Annamaria Izzo, Maria Teresa La Vecchia, Luca Raineri, Patrizia Sabetti, Roberto Sassi, Carmela Servodidio, Nunziatina Sorice, Valentina Cioffi, Angelo Antignani, Giuliana Valerio

**Affiliations:** 1 Italian Federation of Pediatricians Study Center, Naples, 80100 Italy; 2 Benevento Section, Italian Federation of Pediatricians, Benevento, 82010 Italy; 3 Department of Translational Medical Science, Section of Pediatrics, University of Naples Federico II, Naples, 80131 Italy; 4 AOU Federico II, Naples, 80131 Italy; 5 Department of Medical, Movement and Wellbeing Sciences, University of Naples Parthenope, Naples, 80133 Italy

**Keywords:** Antibiotics, Birth weight, Caesarean delivery, Educational level, Household smoking, Parents, Obesity, Overweight, Prevention, Toddlers

## Abstract

**Background:**

Nutrition exerts a fundamental role in the prevention of obesity (OB). The aim of this study was to assess the extent to which well recognized risk factors for early OB can be associated to overweight (OW) or OB under a standardized nutritional approach and surveillance in toddlers.

**Methods:**

The eligible population was represented by 676 toddlers aged 24–36 months, assigned to 18 primary care pediatricians trained on nutritional issues who shared a standardized nutritional approach. Six-hundred-twenty-nine children (333 boys), mean age 27.8 *±* 4.2 months were effectively included in this observational study. Parents received nutritional advice with particular emphasis to proteins and sugar composition supported by leaflets and reinforced at each visit. Body mass index was assessed at the age of 24–36 months. The following individual and family risk factors were considered: gestational age, birth weight, eutocic/caesarean delivery, milk feeding history, household smoking or antibiotics exposure, parents’ weight, height and educational level. Prevalence of OW/OB was compared to a group of 742 toddlers (373 boys) under usual care.

**Results:**

Under a standardized nutritional counselling, 28.1% toddlers were classified as OW/OB compared to 36.9% toddlers under usual care (*p* = 0.005). In unadjusted models, parental OW/OB was significantly associated to OW/OB in toddlers (*p* < 0.01), while high birth weight did not reach statistical significance (*p* = 0.07). In adjusted models, including all the explanatory variables studied, only paternal OW/OB vs. normal weight was significantly associated to OW/OB in toddlers (OR 2.035, 95% confidence interval 1.206–3.436). No protective effect of exclusive breast feeding during the first 6 months of age was demonstrated.

**Conclusions:**

Toddlers under a standardized nutrition counselling focused to limit protein and simple sugars, showed lower prevalence of OW/OB compared to usual care. Healthy promotion activities should take into account the influence of paternal BMI on the offspring adiposity.

## Background

Overweight (OW) and obesity (OB) in children represent public health concerns worldwide, where 41 milions of children under 5 years of age and 340 milions of children and adolescents between 5 and 19 years of age have been reported to be OW or OB, respectively [1].

The last data provided by “Okkio alla salute”, the Italian surveillance system in children aged 8–9 years [2]) showed that the prevalence of OW was 20.4% and OB 9.4%, with higher rates in Southern Italy in 2019. Specifically, the highest prevalence of OW (25.4%) and OB (18.8%) was reported in the Campania region [2].

Several early-life factors significantly contribute to the development of obesity. During the “first 1000 days”, a window of life that includes the pre-conceptional period, maternal influences and nutritional factors play a peculiar role in weight gain compared to other environmental factors, like physical activity and sedentary behaviours. Besides genetic predisposition, high pre-pregnancy body mass index (BMI), increased gestational weight gain and diabetes mellitus are among the main risk factors for early development of obesity [3]. Instead, less explored is the paternal contribution during this early age. With regard to nutritional factors, an important role has been attributed to formula feeding, early age at complementary feeding and increased intake of energy [4]. Specifically, protein intake excess, namely lactoproteins [5, 6] in infants, has been associated to an early adiposity rebound in toddlers, which is predictive of OB persistance and presence of comorbidities later in life [7–9]. Other environmental risk factors for early adiposity, such as type of delivery, birth weight, parental education level, household smoking or early antibiotic therapy have been also explored [3, 10–12].

Prevention strategies of childhood obesity are more feasible and successful in the first years of life. For instance, a systematic review of systematic reviews on the effects of nutritional exposure or interventions in children under 3 years, underscored the promising effect of lowering the protein intake of infant formula or complementary food on the risk of later OW/OB [13]. Indeed, a previous study conducted by three primary care pediatricians from our research group demonstrated that simple but specific dietary advices, focused on the choice and amount of food with low protein content and sugar, were associated to a reduction of the frequency of OW and OB from 26.3 to 13.9% (defined according to the BMI thresholds proposed by the Centers for Disease Control and Prevention) in toddlers living in Campania, an Italian region with high prevalence of OW/OB [14]. Subsequently, this nutritional approach was standardized and implemented among specifically trained Pediatricians working in the Campania region.

As far as we know, there are no studies that compared the association between risk factors for early development of excess weight gain and the prevalence of OW/OB in children under a preventive standardized nutritional counselling.

Therefore, the aim of this prospective study was to assess the extent to which well recognized risk factors for early OB can be associated to overweight (OW) or OB under a standardized nutritional approach and surveillance in toddlers directed at OB prevention.

## Methods

The Regional Health Service provides cost-free health care to all children from birth to 14 years. In particular, each child has a scheduled number of visits from 1 to 144 months, 4 of which from 6 to 36 months of life. Health care is delivered by primary care pediatricians, who are provided with a medical software to store the demographic and clinical data of their child population. This particular organization allowed us to design the following prospective study in the primary care setting. The study was approved by the Ethical Committee “Campania Sud” of the ASL Napoli 3 Sud (protocol number 16, 3/2/2016) and was performed in accordance with the 1975 Declaration of Helsinki revised in 1983. To ensure data protection and confidentiality, data extracted from the medical records were anonimyzed before being uploaded in a database for analyses. Informed consent was obtained by parents of the chidren enrolled in the study.

Briefly, the study involved the entire population of children aged 24–36 months old born from 1 October 2015 to 31 march 2016, under the care of 18 primary care pediatricians working in the Campania region, who participated to an update training about nutrition in the first 36 months of life in order to prevent obesity. After the training, pediatricians shared a protocol of weaning and feeding to be used in all children up to 36 months under their care. Nutrition counselling provided to parents was specifically focused on animal and vegetal protein content from different food sources, avoid abundant portions of food rich in animal proteins, and limit sugar and sugar-sweetened beverages. Leaflets containing practical examples and pictures of portions were delivered according to the different nutrition phases (weaning, 6 to 12 months, 12 to 36 months). The recommended daily protein amount corresponded approximatively to 1.3 gr/kg between 6 and 12 months and 2 gr/kg after 12 months of age; in any case, it was lower than 3.5 gr/kg considered at risk for overweight [15, 16]. The eligible population was represented by 676 children; 47 were excluded for the following reasons: change of residence, change of the registered pediatrician, missing visit at 24–36 months. Therefore 629 children (333 boys, 296 girls), mean age 27.8 *±* 4.2 months were effectively included in the study. Children underwent well-child visits according to the following time sheet: every three months until 12 months of age; every 4 months from 12 to 24 months; every 6 months from 24 to 36 months of age. The well-child visits were increased from 4 (as scheduled according to the Italian Health Service organization) to 7 and they were devoted to assess parents’dietary behaviour and reinforce nutrition advice.

The following individual and family data were available: gestational age, birth weight, type of delivery (eutocic/cesarean), milk feeding history, exposure to household smoking or antibiotics exposure in the first 24 months of life, parents’ weight, height and educational level. Antibiotic exposure was assessed using outpatient prescriptions and patient-reported medications recorded at all primary care visits between birth and 23 months of age. The limit of at least 4 doses during this period was considered a risk factor [17].

Body weight, lenght (under 24 months of age) and height (*≥* 24 months) were measured between 24 and 36 months in each office by the same pediatrician, who was specifically trained in anthropometric standard procedures [18]. Weight was measured on mechanical beam scales, lenght/height were measured to the nearest 0.1 cm using a paidometer or a stadiometer, respectively with the child wearing only underwear and no shoes.

BMI (kg/m^2^) was calculated as body weight divided by the square of height. Categories of normal weight (NW), overweight (OW) and obesity (OB) for children were defined according to the BMI thresholds proposed by the WHO for the children [19], or to the adult cut off of 18.5, 25 and 30 kg/m^2^ for the parents [20]. Parental educational level was categorized as low (elementary and middle school) or high (high school or degree).

Nine primary care pediatricians working in the same region, who did not participate to the nutritional training and did not share the standardized approach, provided anthropometric data of 738 toddlers (373 boys, 365 girls) born in the same period (mean age 27.6 *±* 4.2 months), under their usual care and scheduled number of well-child visits.

### Statistical analysis

Data are expressed as means and standard deviations, medians and interquartile ranges, frequencies (%) and 95% confidence interval (CI). All variables had skewed distribution. Between-groups differences were evaluated by using the Mann Whitney test. Chi-square or Fisher’s exact test, as appropriate, were used to compare proportions. Spearman correlation test was used for correlation analyses. Unadjusted and adjusted binary logistic regression analyses were used and OR with corresponding 95% Confidence Intervals were calculated to evaluate the association of OW/OB status and exposure variables (prenatal/postnatal or family potential risk factors) while controlling for confounding.

A *P* value < 0.05 was considered statistically significant. The statistical analysis was performed using the IBM SPSS Statistics, Version 28.0. Armonk, NY: IBM Corp.

## Results

The overall prevalence of OW/OB in toddlers under standardized care and surveillance was 28.1% (22.1% OW, 6% OB) compared to 36.9% (OW 28.3, OB 8.7%) in children under usual care (*p* = 0.005). Age, BMI-SDS and % of BMI categories did not differ by sex (Table 1).

**Table 1:** Age, BMI-SDS and prevalence of overweight, obesity by sex

	Whole sample	Boys	Girls
Number	629	333	296
Age (months)	27.8 *±* 4.2	27.8 *±* 4.4	27.8 *±* 4.0
BMI-SDS	0.46 (-0.20-1.13)	0.43 (-0.21 + 1.08)	0.48 (-0.19 + 1.14)
Normal weight (number, %)	452 (71.9)	243 (72.7)	211 (71.3)
Overweight/Obesity (number, %)	177 (28.1)	91 (27.3)	85 (28.7)
Overweight (number, %)	139 (22.1)	73 (21.9)	65 (21.9)
Obesity (number, %)	38 (6.0)	18 (5.4)	20 (6.8)

Data are expressed as means *±* standard deviation or medians (95% Confidence Interval)

BMI-SDS, body mass index- standard deviation score

Demographic, anthropometric, prenatal/neonatal or family data were compared between NW and OW/OB toddlers under standardized care and surveillance (Table 2). No differences were found with regard to most variables, except for higher parental BMI values and higher percentage of OW/OB parents in children with OW/OB (Table 2).

**Table 2:** Demographic, anthropometric, prenatal/postnatal and family variables in normal weight and overweight/obese toddlers

	Normal weight *N* = 452	Overweight/obesity *N* = 177	*P*
**Demographic data**			
Boys/girls (numbers)	243/211	91/85	0.762
Age (months)	27.7 ± 4.2	27.9 ± 4.2	0.567
**Anthropometric data**			
Lenght/height (cm)	90.0 (87.0; 93.0)	90.0 (87.8; 94.0)	0.206
Weight (kg)	12.7 (11.8–13.7)	14.8 (13.8–16.0)	< 0.001
BMI (kg/m^2^)	15.9 (15.1; 16.4)	17.9 (17.4;18.5)	< 0.001
BMI-SDS	0.10 (-0.43, 0.58)	1.55 (1.25, 1.92)	< 0.001
**Prenatal/postnatal data**			
Gestational age (weeks) (*n* = 569)	39 (38; 39)	38 (38; 40)	0.239
Birth weight (grams) (*n* = 624)	3,150 (2,895; 3,400)	3,200 (2,940; 3,500)	0.076
Birth weight categories(*n* = 624) low, normal, high (numbers)	36/400/13	7/159/9	0.091
Delivery (eutocic/cesarean) (*n* = 610)	162/277	56/115	0.336
Pregnancy duration (preterm/at term) (*n* = 569)	62/351	18/138	0.288
Breastfeeding at 6 months (yes/not) (*n* = 614)	143/296	60/115	0.684
**Family data**			
Father’s BMI (*n* = 585)	25.8 (23.7; 28.7)	26.4 (24.7; 29.4)	0.005
Mother’s BMI (*n* = 598)	23.7 (21.5; 26.3)	24.5 (22.0; 27.4)	0.008
Father’s BMI status (NW/OW-OB) (*n* = 585)	181/239	51/114	0.007
Mother’s BMI status (NW/OW-OB) (*n* = 598)	279/149	91/79	0.008
Father’s education (low/medium) (*n* = 592)	155/269	70/98	0.248
Mother’s education (low/medium) (*n* = 589)	133/288	52/116	0.880
Household smoking (yes/no) (*n* = 504)	245/115	93/51	0.454
Antibiotic exposure (no/yes) (*n* = 505)	324/48	116/17	0.971

Data are expressed as means *±* standard deviation or medians (95% Confidence Interval)

BMI body mass index, SDS standard deviation score

Correlation analyses showed a slight relationship between BMI z score and birth weight (rho 0.134, *p* < 0.001; father’s BMI (rho 0.148, *p* < 0.001) and mother’s BMI (rho 0.110, *p* < 0.007).

Mother’s BMI was significantly correlated with birth weight (rho 0.124, *p* = 0.002); father s’ BMI (rho 0.237, *p* < 0.001), mother’s education level (rho − 0.134, *p* < 001) and fathers’education level (rho − 0.195, *p* < 0.001). Moreover, father’s BMI was significantly correlated with fathers’education level (rho − 0.141, *p* < 0.001).

The associations between toddlers’ OW/OB status and the different risk factors for OW/OB were explored by logistic regression analyses. The unadjusted and adjusted Odds ratios and 95% CI are synthetized in Table 3. The presence of OW/OB in fathers or mothers (*P* < 0.01) and, at a lesser but not significant extent, high birth weight (*P* = 0.07) were associated with OW/OB in toddlers in the unadjusted model (Model 1). In the adjusted models, caesarean delivery was significantly associated with OW/OB in toddlers compared to vaginal delivery (ORs between 1.696 and 1.788, *p* < 0.01) after controlling for prenatal/neonatal, environmental variables and maternal OW/OB (Models 2–4); the statistical significance disappeared after including the paternal OW/OB variable (Model 5). The presence of OW/OB in fathers was associated with a nearly twofold increase in the association with OW/OB in toddlers, independently of all the prenatal/neonatal, environmental factors, the presence of maternal OW/OB and paternal and maternal education status (Models 5 and 6).

**Table 3:** Associations between toddlers’ status of overweight/obesity and the different risk factors for OW/OB

	Model 1	Model 2	Model 3	Model 4	Model 5	Model 6
Male sex	0.948 (0.669–1.342)	0.875 (0.552–1.387)	0.809 (0.504-1.300)	0.791 (0.491–1.275)	0.817 (0.503–1.326)	0.824 (0.507–1.338)
Preterm birth	0.738 (0.422–1.293)	0.788 (0.375–1.658)	0.780 (0.371–1.640)	0.795 (0.377–1.675)	0.755 (0.356–1.602)	0.738 (0.347–1.569)
Cesarean delivery	1.201 (0.827–1.745)	1.715 (1.044–2.818)*	1.788 (1.083–2.954)*	1.696 (1.023–2.814)*	1.629 (0.977–2.716)°	1.634 (0.980–2.724)°
Normal birth weight	reference	reference	reference	reference	reference	reference
Low birth weight	0.490 (0.201–1.197)	0.590 (0.176–1.974)	0.598 (0.178–2.014)	0.620 (0.184–2.091)	0.642 (0.188–2.196)	0.605 (0.176–2.082)
High birth weight	2.504 (0.959–6.539)°	2.127 (0.532–8.504)	1.970 (0.492–7.894)	1.655 (0.405–6.762)	1.478 (0.357–6.117)	1.544 (0.372–6.410)
No breast feeding at 6 months	0.926 (0.639–1.341)	-	0.797 (0.487–1.304)	0.767 (0.467–1.261)	0.766 (0.464–1.266)	0.765 (0.463–1.266)
Antibiotic exposure	0.989 (0.547–1.789)	-	1.105 (0.563–2.171)	1.040 (0.526–2.056)	0.983 (0.496–1.949)	0.973 (0.490–1.932)
Household smoking	1.168 (0.778–1.755)	-	1.446 (0.883–2.368)	1.387 (0.844–2.280)	1.396 (0.844–2.309)	1.379 (0.828–2.296)
Mothers’ OW/OB	1.626 (1.133–2.333)**	-	-	1.559 (0.967–2.512)°	1.408 (0.866–2.291)	1.390 (0.853–2.263)
Fathers’ OW/OB	1.693 (1.155–2.482)**	-	-	-	2.057 (1.220–3.468)**	2.035 (1.206–3.436)**
Low maternal education	0.971 (0.660–1.428)	-	-	-	-	0.832 (0.481–1.440)
Low paternal education	1.240 (0.861–1.785)	-	-	-	-	1.303 (0.770–2.205)

Model 1 unadjusted; Model 2 adjusted for male sex, preterm birth, cesarean delivery, birth weight (r^2^ = 0.025); Model 3; Model 2 plus no breast feeding at 6 months, antibiotic exposure, household smoking (r^2^ = 0.035); Model 4: Model 3 plus Mothers’ OW/OB (r^2^ = 0.048); Model 5: Model 4 plus fathers’ OW/OB (r^2^ = 0.075); Model 6: Model 5 plus low maternal and low paternal education (r^2^ = 0.079)

*< 0.05 ** < 0.01 ° < 0.07

## Discussion

Our results underline that under a standardized nutritional counselling and surveillance, 28.1% toddlers were classified as OW/OB compared to 36.9% toddlers under usual care. The presence of OW/OB in one or both parents was a significant risk factor in toddlers with OW/OB. After adjustment for all the risk variables, only the presence of OW/OB in fathers was the explanatory variable for OW/OB in toddlers under a standardized nutritional approach.

Pediatric primary care is regarded as an important setting for pediatric obesity prevention and treatment [21, 22]. Pediatricians have an important role in educating parents about specific health behaviors devoted to control energy balance since the very first months of life. In particular, nutrition interventions in the first two years of life can have a positive impact on a child’s BMI, provided that intervention are maintained later in life [23]. Specifically, prospective cohort studies support an association between higher intake of proteins, mainly of animal origin, and increased BMI or BMI z-scores in healthy, well-nourished children from Western countries [24]. Early-life determinants, beginning in the intrauterine life and continuing through the first years of life, may influence the progression of weight gain throughout the life course. However, the efficacy of a nutritional preventive intervention may be weakened by the presence of non modifiable (genetic, epigenetic) or enviromental influences. Our prospective study was designed to assess the extent to which well recognized risk factors for early obesity characterize toddlers with OW/OB under a standardized nutritional counselling and surveillance.

Our results underline that the presence of OW/OB in one or both parents was a significant risk factor in toddlers with OW/OB in unadjusted analyses. In particular, the OR for OW/OB was a 1.6 times higher whether either fathers or mothers were affected by OW/OB compared to their normal weight conterpart, confirming previous results. The strict association berween parental obesity and early childhood obesity can be interpreted as a consequence of the complex interplay among genetic, epigenetic and environmental factors. By comparing the strenght of the association of BMI between parents and their offspring, several Authors reported that this association was stronger in mothers, when the birth weight was considered; in this case the reason may be ascribed to the intrauterine environment related to maternal BMI [25, 26]. On the contrary, associations between parents and offsprings adiposity did not differ between fathers and mothers when children were measured at later ages [27, 28]. In particular, a previous cohort study performed in the Netherlands found a similar association of both paternal and maternal BMI with preschool overweight, after controlling for 34 putative parental, fetal, and infant factors with overweight risk [29]. At variance with this latter study, we found that the association of OB status between the parent-child dyad disappeared when the mothers were considered, after controlling for several risk factors that are associated with maternal obesity, while it persisted in the fathers. Indeed, maternal OB is linked to greater risk of preterm birth, large-for-gestational-age babies or lower breastfeeding rates [30] thus probably explaining why the association was not confirmed in the adjusted models. Our findings underline the independent role of paternal BMI on children’s adiposity in the very firs years of life, that can be supported by genetic and epigenetic influences [31] and/or family environment about parenting practice. Despite food parenting is most often guided by mothers in the first 2–3 years of age, we cannot exclude that nowadays fathers may directly influence feeding practice in different ways and levels during the first few years of life [32].

We did not find any influence of low birth weight on the prevalence of OW/OB in our cohort; indeed, this association has been usually reported after the age of 8 years [16]. Other determinants, such as large birth weight or preterm delivery did not appear to be associated to the risk of OW/OB in our cohort.

Increasing evidences points to cesarean delivery as a possible risk factor for obesity, but the underlying mechanism of this association remains unclear [33, 34]. Of note, we found that the OR of OW/OB in toddlers born by caesarean compared to vaginal delivery was significant as long as parental OW/OB status was not included to the model, thus explaining that the impact of caesarean delivery is reduced by adding stronger risk factors. The effect of cesarean delivery on children’s obesity has been reported to be higher in places where the cesarean delivery rate was > 10% (RR = 1.30, 95% CI: 1.10–1.50) [35]. Interestingly, the rate of cesarean births in 2021 in the Campania region was the highest in Italy (50.2% vs. 31.2%) as well as the prevalence of OW/OB [2, 36], therefore this possible risk factor deserves further research. Indeed, other studies have shown that the association between caesarean delivery and OW/OB may be invalidated by further subgroup analyses. For instance, Li et al. [37] found that the overall OR for OW/OB after cesarean delivery was 1.32 (95% CI 1.15–1.51) in children berween 3–8 years of age, but it remained significant only for OB (OR 1.40, 95% CI 1.17, 1.67) and not for OW (OR 1.22, 95% CI 0.99, 1.50). Furthermore, a prospective study performed in United States reported an unadjusted OR for OB of 2.40 (1.60 to 3.62) in children at 3 years of age born from caesarean delivery, that decreased to 2.10 (1.36, 3.23) when adjusted for several child (age, sex and birth weight) and maternal predictors (age, education, race/ethnicity, prepregnancy BMI). Of note, this association was significant only in mothers with normal pre-pregnancy weight [38].

Our study does not support that excludive breast-feeding up to six months protects from OW/OB, nor that antibiotic exposure or household smoking are significant risk factors for it. Concerning breastfeeding, we cannot exclude that more prolonged periods of breastfeeding while children received nutritionally adequate and safe complementary foods might have exerted a protective role in our setting.

Our study has some weaknesses: parents’BMI was based on self-reported height and weight, and individual and family data of the control group were lacking. The strenght of our study is the large number of trained pediatricians, who provided periodically health visits and nutritional education to a large sample of toddlers and families in the first 24–36 months of life. In addition, the inclusion of a variety of prenatal, postnatal and family risk factors for early obesity, which are frequently interrelated each other, allowed us to perform statistical analyses adjusted for several confounders.

## Conclusions

Our study performed in a region with the highest prevalence of OW/OB in children highlights that nutritional counseling focused on protein and sugar intakes during the first years of life should be implemented among Pediatricians working in a primary care setting. Healthy promotion activities should take into account the influence of paternal BMI on the offspring adiposity.

## Data Availability

The datasets used and/or analyzed during the current study are available from the corresponding author on reasonable request.

## Abbreviations

BMI: Body Mass Index
CI: Confidence Interval
OB: Obesity
OW: Overweight
SDS: Standard Deviation Score
